# Twisted Mesenteric Leiomyoma Mimicking Acute Appendicitis

**DOI:** 10.7759/cureus.25260

**Published:** 2022-05-23

**Authors:** Mohammad Izwan Mohd Isa, Andee Dzulkarnaen Zakaria, Wan Zainira Wan Zain, Wan Suriza Wan Ab Rahman, Junaidi Awg Isa

**Affiliations:** 1 Department of Surgery, Universiti Sains Malaysia, School of Medical Sciences, Kota Bharu, MYS; 2 Department of Surgery, Hospital Universiti Sains Malaysia, Kota Bharu, MYS; 3 Department of Surgery, Hospital Kuala Lumpur, Kuala Lumpur, MYS; 4 Department of Surgery, Hospital Raja Perempuan Zainab II, Kota Bharu, MYS

**Keywords:** segmental resection and anastomosis, laparoscopy, acute appendicitis, leiomyoma, infarcted mesenteric tumour

## Abstract

Leiomyoma is a benign tumor of smooth muscles and can essentially arise from any part of the body that contains smooth muscles. It is more commonly found in the uterus, while those from the gastrointestinal tract are rare. The spectrum of clinical presentation is rather wide, from a vague abdominal discomfort or acute abdomen mimicking more common diagnoses such as acute appendicitis or acute gastroenteritis to features of intestinal obstruction and gastrointestinal bleeding. Here, we share our experience with a case of a young woman whose symptoms and signs were typical of acute appendicitis, which intraoperatively turned out to be a twisted mesenteric tumor located at 70 cm from duodenojejunal flexure. The tumor was managed by segmental resection and anastomosis. She had an uneventful recovery postoperatively, and histopathological examination of the surgical specimen was consistent with an infarcted mesenteric leiomyoma. The rarity of this entity mimicking appendicitis is highlighted, and should it never get twisted, it would have probably remained unnoticed for the rest of her life. In the current era of laparoscopy, it is always wise to never skip a thorough examination of the whole length of the small bowel in order to locate this type of pathology.

## Introduction

Primary mesenteric tumors are rare and are usually of mesenchymal origin. In fact, mesentery is more commonly associated with metastatic deposits. Benign tumors include leiomyoma, lipomas, desmoid tumors, and cystic lymphangiomas, while malignant ones include lymphoma, gastrointestinal stromal tumors (GIST), carcinoids, and leiomyosarcomas [1]. The rarity of this group of tumors reported in the literature explains why specific management guidelines are scarce, and the treatment is usually individualized. In addition to this rarity, the diversity of their clinical features also makes them difficult to diagnose, some are incidentally found during CT of the abdomen, and some even mimic more common surgical diagnoses such as acute appendicitis. As a result, the clinical management is that of acute appendicitis, and therefore, most mesenteric tumors are detected during the surgery. This usually ends up with unplanned bowel resection, provided the tumor is amenable to resection. Here we share our experience managing a 25-year-old woman with mesenteric leiomyoma who presented to us with features of acute appendicitis.

## Case presentation

A 25-year-old female with no underlying medical illness presented to us with three-day right iliac fossa pain. She presented on day 3 of illness as the pain was getting more intense; her pain score was 9/10. The pain was colicky in nature and had a migratory character. It began at the left iliac fossa before migrating to the periumbilical region and eventually localized to the right iliac fossa. It was associated with nausea but no vomiting. Menstrual and gynecological history was otherwise unremarkable. Clinical examination revealed normal vital signs. Abdominal examination revealed tenderness at the right iliac fossa with no guarding and no rebound tenderness. Rovsing's sign was negative, and there was no palpable abdominal mass. Gynecological examination with transabdominal scan was normal. With normal urine full examination and microscopy examination (FEME), presence of leucocytosis, and Alvarado score of 7, a diagnosis of acute appendicitis was made. She was then booked for laparoscopic appendicectomy.

**Figure 1:**
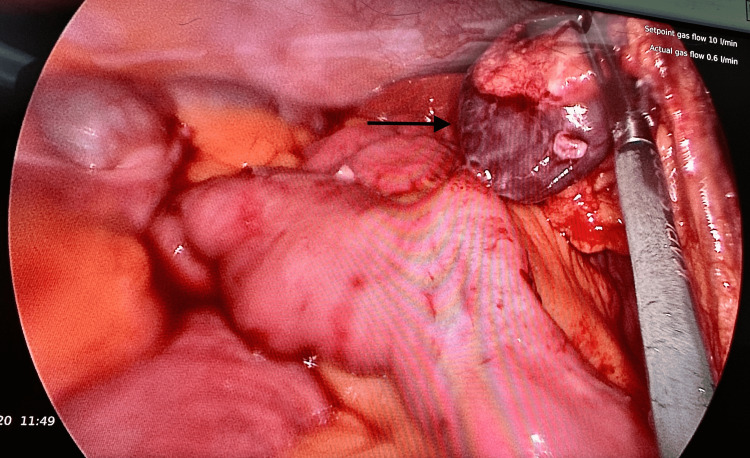
Laparoscopy shows a twisted and infarcted mesenteric tumor (arrow) located at 70 cm from duodenojejunal flexure

On laparoscopy, clumping of omentum was noted centrally. Examination of the appendix, fallopian tubes, ovaries, and uterus were all normal. The initially seen omental clump was then dismantled, after which a gangrenous pedunculated swelling arising from the mesenteric border of jejunum was revealed. Its surface was regular and smooth, firm in consistency, but showed adhesions with the omentum. The swelling was 5 x 3 cm in diameter and twisted at its pedicle (Figure 1). No significant narrowing of the adjacent small bowel was noted. This pathology was located at 70 cm from duodenojejunal flexure. The rest of the small bowel was normal, with no mesenteric lymph nodes found. Liver, gall bladder, stomach, and colon were normal. Due to technical limitations, the procedure was converted to midline laparotomy. Oncologic resection was performed with a 10 cm margin, and primary anastomosis was performed. Postoperatively, she had an uneventful recovery. She resumed her normal diet on postoperative day 2 and was discharged home on postoperative day 4 with a clean wound.

On histopathological examination, the swelling showed an extensive area of hemorrhage with scattered blood vessels and neutrophil infiltration. It was well circumscribed and composed of a few viable spindle cells at the periphery, which were uniform, showing oval vesicular nuclei with wavy indistinct cytoplasm. These cells were positive for smooth muscle actin (SMA) while negative for CD117, S100, and CD34. This swelling was connected to the outer serosal surface of the small bowel. The resected small bowel showed normal histomorphology. This was finally concluded as normal small bowel with an attached infarcted smooth muscle tumor, suggestive of mesenteric leiomyoma. Two months after the surgery, the patient was reassessed in the clinic. She was well and had no symptoms. Considering the benign nature of the tumor and adequate resection performed, she was discharged from the surgical clinic.

## Discussion

Leiomyoma is a benign tumor that arises from smooth muscles. They are most commonly found in the uterus and may also arise elsewhere in the body, including the gastrointestinal tract and mesentery. It is important to distinguish them from leiomyosarcoma and other types of mesenchymal tumors such as GIST. By immunohistochemistry, both tumors are positive for SMA, but leiomyoma is negative for CD117, CD34, and S100. In smooth muscle tumors, the presence of atypia and high mitotic activity indicates their malignant nature, leiomyosarcoma [2-4]. This differentiation is important as it affects the long-term management and prognosis. In GIST, treatment with a tyrosine kinase inhibitor is mandated to prevent a recurrence, while in leiomyoma, surgical resection alone would be curative [1]. The extent of resection is guided by the location of the mass. Well-circumscribed mass with pedicle may be removed with simple excision, but those at the mesenteric border or on the small bowel require segmental resection and anastomosis. Gupta et al. described a retrovesical lesion that turned out to be arising from small bowel mesentery. Its retrovesical location was confirmed radiologically prior to the surgery. The mass was excised by clipping at its pedicle followed by division, and histopathological examination confirmed a benign leiomyoma [5].

As a matter of fact, mesenteric and small bowel tumors tend to have a similar clinical presentation. They may manifest as small bowel obstruction, gastrointestinal bleeding, abdominal mass, a wide spectrum of abdominal pain, or even a small bowel intussusception [6-7]. Obstruction or bleeding usually prompts the clinician to urgently investigate and intervene; however, abdominal pain, especially the vague one, without any obvious clinical signs and normal routine investigation, tends to mislead the clinician. On the other hand, the abdominal pain may mimic certain common clinical conditions such as acute appendicitis, salpingitis, or gastroenteritis, and thus subsequent therapy is administered according to such diagnosis. Our case is one of those examples that presented the clinical picture of acute appendicitis. Literature search on surgical conditions mimicking acute appendicitis tend to be of small bowel or mesenteric origin, and among the pathologies reported were small bowel leiomyoma, ruptured jejunal GIST, and ruptured desmoid tumor [8-10]. In addition, a case of mesenteric leiomyoma with synchronous uterine leiomyoma was also reported [11].

In our case, the migratory nature of the abdominal pain, which eventually resided in the right iliac fossa, presence of right iliac fossa tenderness, normal gynecological assessment, and exclusion of urinary tract infection were all positive points that led us to a diagnosis of acute appendicitis. The clinical diagnosis was convincing enough to proceed with laparoscopic appendicectomy. Of note, we do not routinely perform an ultrasound or CT scan to establish a diagnosis of appendicitis in young adults with a clinically convincing presentation. Retrospectively, it would have been a better move to have a CT of the abdomen performed before the surgery. CT would be appropriate, especially in establishing other causes of right iliac fossa pain, such as appendicular abscess, which can be drained extraperitoneally, and it would aid in the diagnosis of the mesenteric tumor. With this, better preoperative planning would have been undertaken. During the surgery, the rather surprising intraoperative finding was dealt with an oncological resection with a 10 cm margin and primary anastomosis by taking into consideration that a malignant tumor was still a possibility. This procedure is best performed laparoscopically if the expertise is available, and this, in fact, would promote faster recovery. We concluded that her pain was explained by twisting and infarction of her mesenteric tumor. She never had vomiting because there was no obstructing effect by the tumor. It is also worth noting that laparoscopy has a significant advantage in localizing the pathology, especially when the appendix appears normal and, more importantly, when the common alternative diagnosis is not found. A thorough search for the pathology is a must before concluding a negative laparoscopy. On a side note, it would have been challenging to localize the pathology if this case had been performed using the conventional open approach via Lanz incision.

## Conclusions

In conclusion, clinicians should be aware of the uncommon differential diagnosis of acute appendicitis. As the mimics usually originate from the small bowel and mesentery, the whole length of the small bowel and its mesentery must thoroughly be inspected. Since the differentiation between malignant and benign mesenteric tumors is difficult intraoperatively, resection should be performed following the principles of resection of malignant disease. Diagnostic laparoscopy has been a game-changer as it can readily show the exact pathology, especially when the appendix is normal, or synchronous pathology when the appendix is abnormal. Thorough diagnostic laparoscopy is a key to not missing a rare pathology such as mesenteric tumors.
